# Changes in the Visual Field Test after Descemet Stripping Automated Endothelial Keratoplasty in Advanced Glaucoma

**DOI:** 10.3390/jcm13051431

**Published:** 2024-03-01

**Authors:** Noriko Toyokawa, Kaoru Araki-Sasaki, Hideya Kimura, Shinichiro Kuroda

**Affiliations:** 1 Nagata Eye Clinic, 1147 Kitayamada Horai, Nara City 631-0844, Nara, Japan; noritama0620@topaz.ocn.ne.jp (N.T.); hkimura@pearl.ocn.ne.jp (H.K.); zzkuroda@skyblue.ocn.ne.jp (S.K.); 2 Department of Ophthalmology, Kansai Medical University, 2-5-1 Shinmachi, Hirakata City 573-1010, Osaka, Japan

**Keywords:** DSAEK, glaucoma, visual field test, Goldmann perimetry, Humphrey Field Analyzer

## Abstract

**Background**: To evaluate changes in the visual field (VF) after Descemet stripping automated endothelial keratoplasty (DSAEK) in eyes with advanced glaucoma and previous trabeculectomy. **Methods**: Changes in VF, best-corrected visual acuity (BCVA), intraocular pressure (IOP), and number of glaucoma medications were analyzed before and after DSAEK in 19 eyes. The VFs were evaluated using the 10-2 program of the Humphrey Field Analyzer (HFA) and/or Goldmann perimetry (GP). **Results**: In nine eyes, the MD improved from −22.24 ± 6.5 dB to −18.36 ± 5.1 dB in HFA. In five out of nine eyes, postoperative MD improved >1 dB compared to preoperative MD. In GP testing, 10 out of 15 eyes showed an improvement, that is, greater than 20° in VF enlargement by the isopter of I-4e and/or new detection of a smaller or darker isopter. Overall, improvement in VF with the HFA and/or GP test was observed in 12/19 (63.2%) eyes after DSAEK. Postoperative BCVA improved by more than two lines in logMAR VA in 18 of 19 (94.7%) eyes. There were no significant differences between the preoperative and postoperative IOP and the number of glaucoma medications. **Conclusions**: DSAEK may produce subjective improvement in the visual field as well as improved visual acuity, even in advanced glaucomatous eyes.

## 1. Introduction

Descemet stripping endothelial keratoplasty (DSAEK) has been reported as a feasible technique for compensation of corneal endothelial dysfunction due to glaucoma [1,2,3,4]. However, previous glaucoma filtration surgery, such as trabeculectomy or glaucoma drainage device implantation, is a significant risk factor for graft failure [2,3,4,5,6,7,8,9]. According to a previous report, the 5-year graft survival rate was 59% or 39% in glaucomatous eyes with previous trabeculectomy [5,9]. This is clearly lower than 73% in eyes without glaucoma, 100% in eyes with primary angle closure disease, and 80% in glaucomatous eyes without previous trabeculectomy [9]. Thus, the authors mentioned that previous trabeculectomy was significantly associated with endothelial cell loss and concluded that prior glaucoma surgery was a significant risk factor for corneal endothelial failure after DSAEK [5,9].

A history of glaucoma drainage device use also drastically reduces the number of endothelial cells after DSAEK [7]. Kang et al. reported that graft dislocation occurred in 35.7%, early graft failure developed in 15.5% and secondary graft failure developed in 22.5% after DSAEK in eyes that had undergone previous glaucoma surgery [10].

In addition to these complications, visual function improvement, including visual acuity and visual field (VF), after DSAEK remains a concern, especially in advanced glaucoma because of severe VF defects. Thus, the indication for transplantation in advanced glaucoma is always difficult to identify. Regarding the visual prognosis, visual acuity after DSAEK in glaucomatous eyes has been reported [2,6,7,11]. However, changes in the VF test before and after DSAEK in patients with advanced glaucoma have not been reported. The aim of the present study was to evaluate changes in the VF test before and after DSAEK in eyes with previous trabeculectomy due to advanced glaucoma to assist in determining surgical indications for DSAEK.

## 2. Materials and Methods

The study protocol was approved by the Nagata Eye Clinic Institutional Review Board, and written informed consent was obtained from all patients, according to the Declaration of Helsinki.

We performed a retrospective chart review of all consecutive patients who had received DSAEK for bullous keratopathy in advanced glaucoma eyes with previous trabeculectomy from February 2014 to February 2021 at the Nagata Eye Clinic. Patients with under 6 months of observation periods after DSAEK and no results of VF tests before and after DSAEK were excluded from the study.

### 2.1. Surgical Procedures and Postoperative Management

Two experienced surgeons (KAS and SK) performed all of the procedures. The general surgical technique was based on currently accepted standard practice. Compression sutures were placed in cases with large and thin conjunctival wall blebs. A peripheral iridectomy was performed in the inferior part of the iris to prevent postoperative pupillary block. The graft was inserted using a pull-through technique with the Busin glide (Moria, Doylestown, PA, USA). First, a pupil-sized amount of air was injected into the anterior chamber, and the graft was centered using the Ambler LASIK/DSAEK roller (Ambler Surgical, Exton, PA, USA). Then, a sufficient amount of additional air was injected into the anterior chamber. Intraoperative manipulation of goniosynechialysis was minimized to prevent excessive postoperative inflammation. IOP was measured two hours postoperatively to confirm that it remained within the normal range.

Topical drops of levofloxacin 0.5% (Cravit; Santen, Osaka, Japan) and betamethasone 0.1% (Rinderon; Shionogi, Osaka, Japan) were prescribed five times daily, starting a day after the surgery. Atropine sulfate hydrate eye ointment 1% (Ryuato; Santen, Osaka, Japan) was applied, as needed, to avoid the pupillary block. Levofloxacin was discontinued approximately 2–3 weeks after surgery or until the corneal endothelium healed. The topical steroid dose was tapered to once daily over a period of approximately 4–6 months. Topical glaucoma medication was restarted as needed postoperatively.

### 2.2. Intraoperative Complications

No significant intraoperative complications, such as posterior dislocation of the donor DSAEK button, intraocular hemorrhage, or suprachoroidal hemorrhage, developed.

### 2.3. Data Collection and Analysis

Postoperative demographics, ocular history, previous surgery, glaucoma history, and intraoperative and postoperative outcomes were reviewed retrospectively from the patient charts. A paired *t*-test was used for pre- and post-operative comparisons of IOP and mean deviation (MD). The Wilcoxon signed-ranks test was used for pre- and post-operative comparisons of the number of glaucoma medications and visual acuity. A Kaplan–Meier survival analysis was performed to evaluate graft survival. Distal visual acuities were converted to the logarithm of the minimal angle of resolution (logMAR) for statistical analysis. Very low visual acuities such as “hand motion” and “counting fingers” were converted to LogMAR 2.3 and LogMAR 2.0, respectively [12]. Changes in visual acuity postoperatively (improvement or worsening) were defined as a >2-line increase or decrease in logMAR visual acuity. IOP was measured using an iCare-bound tonometer (Icare, Finland Oy, Vnataa, Finland) or a Goldmann applanation tonometer (Haag-Streit, Koeniz, Switzerland). The data were analyzed using StatMate V (AtmsCorp, Tokyo, Japan). All *p*-values < 0.05 were considered statistically significant.

### 2.4. Evaluation of the Visual Field

The VF was evaluated using a Humphrey Field Analyzer (Carl Zeiss-Meditec, Dublin, CA, USA) and/or a Goldman perimetry (model S-940, Haag-Streit, Berne, Switzerland). Preoperative VF tests were performed after the diagnosis of bullous keratopathy.

(a) Humphery field analyzer (HFA)

Patients underwent HFA with a 10-2 test pattern using the Swedish Interactive Thresholding Algorithm (SITA) standard. The mean deviation (MD) was compared before and after surgery using the paired *t*-test. An increase in MD > 1.0 dB was defined as an improvement in the HFA test and a decrease in MD < 1.0 dB as a worsening.

(b) Goldman perimetry (GP)

The following findings were stipulated to suggest improvements in the GP test: (1) greater than 20° of enlargement of the VF plotted by the I-4e isopter, determined by the total degrees of eight directions, including upper, temporal upper, temporal, temporal lower, lower, nasal lower, nasal upper, and nasal directions; and/or (2) new detection of the smaller or darker isopter in the GP test after DSAEK.

### 2.5. Definition of Advanced Glaucoma

Glaucomatous VF abnormalities in both hemifields, and/or loss within 5° of fixation in at least one hemifield [13].

## 3. Results

### 3.1. Patient Demographics

Table 1 summarizes the patient demographics, including the type of underlying glaucoma. Nineteen eyes of nineteen patients with advanced glaucoma, aged 73 ± 11 years, who had undergone DSAEK were included in this retrospective study. There were 14 men and 5 women. All patients had undergone trabeculectomy 70.6 ± 57.2 months earlier, before DSAEK, and the average number of filtration surgeries was 1.3 ± 0.6. The mean follow-up period after DSAEK was 27 ± 18 months. All the eyes had advanced glaucoma and pseudophakic. IOP was controlled with or without glaucoma medication in all eyes prior to DSAEK. Concurrent procedures during the DSAEK included compression sutures on the bleb in four eyes. Rebubbling was required in one eye, which resulted in successful attachment.

### 3.2. Visual Acuity

The mean preoperative logMAR visual acuity was 1.6 ± 0.6, and it significantly improved to 0.8 ± 0.5 (*p* < 0.05). Overall, visual acuity in 18 of 19 (94.7%) eyes had improved by two lines at 1 year postoperatively compared to that recorded preoperatively.

### 3.3. Postoperative IOP Control and Graft Outcomes

The mean preoperative IOP was 9.5 ± 4.4 mmHg, and the mean postoperative IOP was 10.6 ± 4.2 mmHg. The mean number of preoperative and postoperative topical glaucoma medications was 0.8 ± 1.3 and 1.1 ± 1.5, respectively. There were no significant differences (*p* > 0.05) between the preoperative and postoperative IOP and the number of glaucoma medications. The Kaplan–Meier graft survival rate at 1 year was 89%.

### 3.4. Visual Field Test

HFA was performed in 9 cases and GP in 15 cases, of which 5 cases (cases 1, 2, 3, 4, and 5; Table 2 and Table 3) had both tests performed. Preoperative VF testing was performed for 4.7 months on average (range: 1–12 months) before DSAEK. A postoperative VF test was performed 7.8 months on average (range: 2–12 months) after DSAEK.

In HFA, the preoperative MD of −22.24 ± 6.5 dB improved to the postoperative MD of −18.36 ± 5.1 dB, although this is not a significant difference (*p* = 0.07). However, in five out of nine eyes (55.6%), postoperative MD improved by more than 1 dB after DSAEK. Three remained unchanged (case 4, 5, 6), that is, within 1 dB change, and the remaining one eye (case 7) worsened after DSAEK. (Table 2)

Table 3 shows the results of the GP test. Ten (66.7%) out of fifteen eyes showed an improvement of greater than 20° of enlargement of the VF of I-4e isopter and/or new detection of the smaller or darker isopter after DSAEK. The remaining four eyes (case 4,12,13,19) showed no changes. There was only one case in which GP was worsened after DSAEK (case 18).

Overall, 12/19 (63.2%) eyes improved in VF with HFA and/or GP tests after DSAEK. Among them, three eyes showed an improvement both in HFA and GP testing. Patients who deteriorated on HFA or GP did not have any complications such as IOP elevation during and even after the operation.

### 3.5. Representative Cases

**Figure 1:**
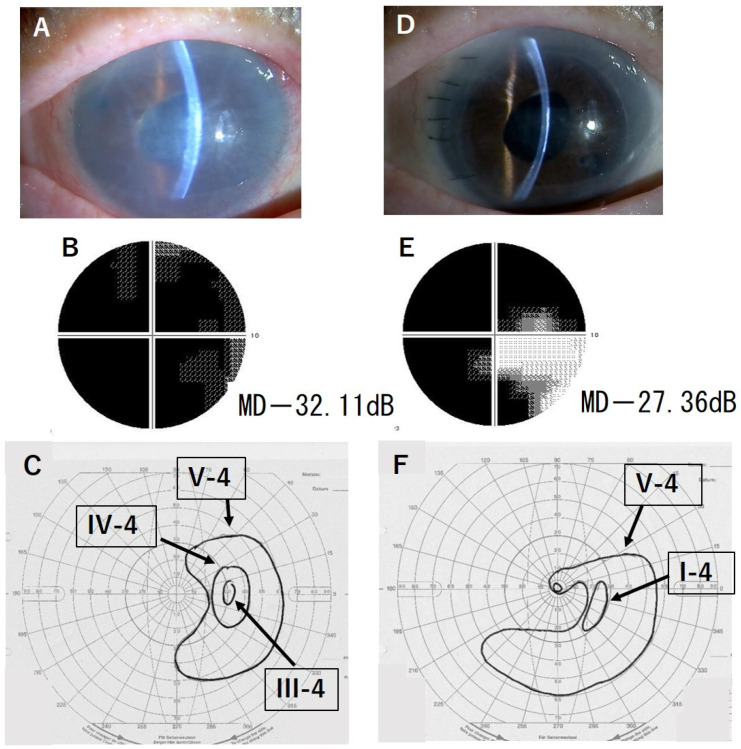
Case 1, 86-year-old female. (**A**): Slit-lamp photograph before DSAEK, LogMAR BCVA 1.2. (**B**): HFA 10-2 test before DSAEK. (**C**): GP test before DSAEK, no central visual field. (**D**): Slit-lamp photograph after DSAEK, LogMAR BCVA 0.4. (**E**): HFA 10-2 test after DSAEK. (**F**): GP test after DSAEK, the central visual field was newly detected, and the peripheral visual field was enlarged. BCVA: best-corrected visual acuity; HFA: Humphrey Field Analyzer; MD: mean deviation. GP: Goldmann perimetry. DSAEK: Descemet stripping automated endothelial keratoplasty. MD: mean deviation.

**Figure 2:**
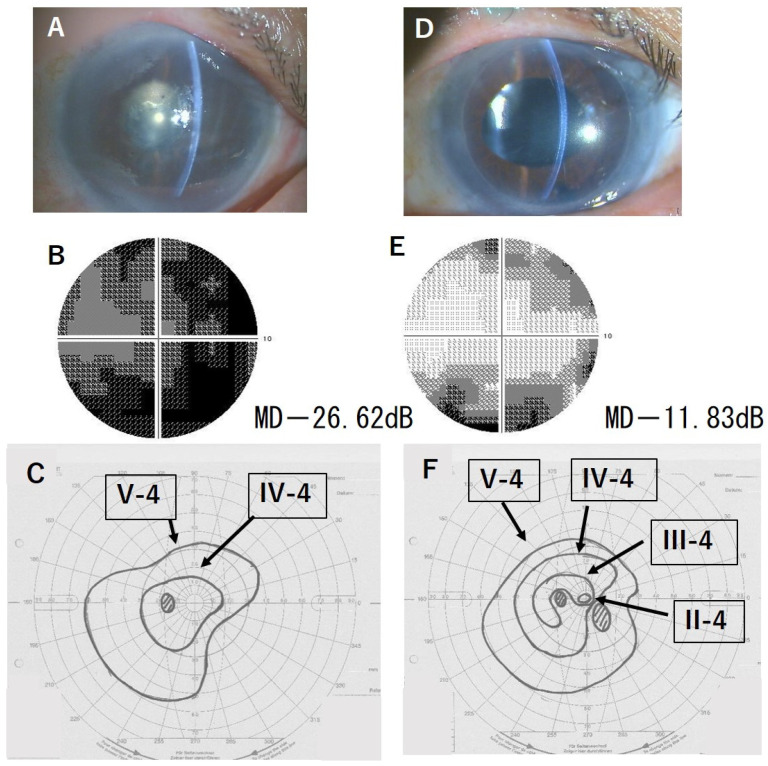
Case 2, 78-year-old male. (**A**): Slit-lamp photograph before DSAEK, LogMAR BCVA 1.7. (**B**): HFA 10-2 test before DSAEK. (**C**): GP test before DSAEK. (**D**): Slit-lamp photograph after DSAEK, LogMAR BCVA 0.5. (**E**): HFA 10-2 test after DSAEK. (**F**): GP test after DSAEK, the central visual field by the smaller isopter was newly detected. BCVA: best-corrected visual acuity; HFA: Humphrey Field Analyzer; MD: mean deviation. GP: Goldmann perimetry. DSAEK: Descemet stripping automated endothelial keratoplasty.

Figure 1 and Figure 2 show two representative cases of improved VF test results. In these two cases, no central VF was detected before DSAEK. However, after DSAEK, not only was the peripheral VF enlarged, but also, the central VF appeared.

## 4. Discussion

We have shown that DSAEK for advanced glaucoma with a history of previous trabeculectomy restored the visual field in 63.2% of patients. We speculate that this is not because of the recovery of optic nerve function but improved visibility due to restoration of corneal transparency. We assume that the optic nerve did not improve functionally, but rather that there was a subjective enlargement of the VF. In advanced glaucoma, corneal opacity may affect subjective VF more severely rather than the actual VF defect. It is difficult to precisely evaluate a glaucomatous VF defect in eyes with bullous keratopathy. When a patient is suffering from bullous keratopathy, the VF defects of glaucoma may be assessed as more severe than they actually are. The keratoplasty surgeon should be aware of this underestimation of VF when considering the indication for DSAEK, because there are some situations in which the surgeon gives up on the operation because of a narrowness of the visual field.

It is known that a decline in sensitivity in the central inferior area of the VF has the strongest association with longitudinal vision-related quality of life in patients with glaucoma [14,15,16], since severe glaucomatous VF defects are associated with disability throughout daily activities [17]. Even small improvements in VF can be meaningful for patients with bilateral advanced glaucoma, as shown in cataract surgery [18]. The fact that in our study, glaucoma patients with advanced narrow visual fields with a history of trabeculectomy had visual field expansion after DSAEK suggests that DSAEK should not be given up as totally inapplicable in these patients.

On the contrary, two eyes had worsened VF after DSAEK, despite the well-controlled IOP. These eyes had no complications during or after DSAEK. Although the surgical invasion of DSAEK might lead to fluctuations in the IOP during the surgery, we speculate that IOP control within the normal range after DSAEK is sometimes insufficient in the advanced stage of glaucoma [19,20]. Although corneal thickness changes after DSAEK, this has been reported to have no effect on IOP values measured with Tonopen XL or Goldmann applanation tonometer after DSAEK [21,22].

The incidence of post-DSAEK ocular hypertension was reported as 51.9%, including steroid-induced IOP elevation [23]. The authors also found that pseudophakia (*p* = 0.024) and preoperative IOP > 16 (*p* = 0.003) were risk factors of post-DSAEK ocular hypertension. All of our advanced glaucoma cases with multiple glaucoma filtration surgeries were pseudophakia, including some with IOPs of 16 mmHg or higher. This indicates that our cases were at risk of elevated postoperative intraocular pressure and graft failure. However, it was reported that the postoperative IOP elevation rate was higher in penetrating keratoplasty (50%) than that in DSAEK (19.6%) [4]. And so, we decided that DSAEK was preferable to PKP for our patients with bullous keratopathy with advanced glaucoma. In fact, none of our cases were difficult to deal with due to elevated postoperative intraocular pressure and there was no deterioration of the visual field in most cases.

Even though DSAEK has many advantages over PKP, there are still other concerns such as pupillary block and peripheral anterior synechiae. We created an intraoperative iridectomy in all cases and thus could avoid the pupillary block.

An additional complication with previous trabeculectomy is air escaping through the sclerostomy site of trabeculectomy. We could also avoid this complication by adding compression sutures on the bleb. Thus, we tried to perform DSAEK with special care to avoid such kinds of complications. Another report indicates that graft detachment is likely to occur in DSAEK after trabeculectomy, but this is not related to graft failure [24]. Besides, epithelial downgrowth between 3 months and 4 years postoperatively and intraocular lens dislocation were reported as the complications of DSAEK [25,26,27], and these complications require additional surgical procedures. However, we experienced none of these complications in our cases.

Although the current study showed 63.2% improvement in the VF test after DSAEK, previous studies reported poor graft survival in eyes with prior glaucoma filtration surgery [2,3,4,5,6,7,8,9]. In a study of as many as 130 eyes, the overall graft survival rate 5 years postoperatively was 85%, but it was less than 50% in cases with bleb, such as post-trabeculectomy [28]. This low 5-year survival rate might be related to the environment within the anterior chamber of glaucoma, [29,30], especially with bleb. When considering the indication for DSAEK, the graft survival rate should be considered comprehensively along with the prognosis of VF. Of course, excessive medical treatment and excessive expectations should be avoided, and the patient’s background, wishes, and other factors should be discussed with the patient and the data presented to determine the appropriateness of the procedure. However, according to our results, there seems to be no reason why a patient should be excluded from DSAEK because of an advanced visual field after trabeculectomy.

This study has several limitations, such as its retrospective design, obviously small sample size, and lack of standardization of the timing of the VF test. Regarding GP testing, there are technical limitations. The result of the GP test may be subjective and less reproducible. In addition, multiple VF tests are necessary to evaluate accurate changes in VF. However, these were not performed to avoid patient burden. Regarding the VF program, HFA 10-2 program is suitable for patients with advanced glaucoma. However, for some patients with advanced glaucoma and bullous keratopathy, only GP was available. A further prospective study is necessary, and should include patient satisfaction surveys. We need to recruit a larger number of people, add a central visual field by HFA, and establish a method to collect prospective data to study.

To summarize, in a study involving patients with advanced glaucoma, 63.2% of patients achieved improvement in the VF test, and 94.7% of patients experienced improved BCVA after DSAEK. We believe that the present data will be useful in determining the indication for DSAEK in eyes with advanced glaucoma and bullous keratopathy.

## 5. Conclusions

In conclusion, 63.2% of patients achieved improvement in the VF test and 94.7% of patients experienced improved BCVA after DSAEK in patients with advanced glaucoma.

We believe that the present data will be useful in determining the indication for DSAEK in eyes with advanced glaucoma and bullous keratopathy.

## Figures and Tables

**Table 1:** Patient demographics including the type of underlying glaucoma.

Characteristics	Ratio/Percentage/Stages
Sex (male–female)	14:5
Age at the time of DSAEK (years)	73 ± 11 (mean ± SD)
Type of glaucoma Primary open-angle glaucoma including normal tension glaucoma Exfoliative glaucoma Secondary open-angle glaucoma	7 (36.8%) eyes Normal tension glaucoma in 1 eye 6 (31.6%) eyes 6 (31.6%) eyes
Lens status	Pseudophakic in all eyes
Glaucoma severity	Advanced in all eyes

SD: standard deviation; DSAEK: Descemet stripping automated endothelial keratoplasty.

**Table 2:** Changes in visual acuity and MD value in Humphrey Field Analyzer with a 10-2 test.

Case	BCVA (logMAR)		VF MD (dB)	VF Improvement	
	Preoperative	Postoperative	Preoperative	Postoperative	(Difference in Value)
1	1.2	0.4	−32.11	−27.36	I (+4.75)
2	1.7	0.5	−26.62	−11.83	I (+14.75)
3	2.0	0.15	−28.03	−17.25	I (+10.78)
4	1.4	0.7	−21.06	−20.36	N (+0.7)
5	2.3	0.7	−25.80	−25.36	N (+0.44)
6	0.5	0.3	−12.81	−13.77	N (−0.96)
7	1.1	0.4	−13.95	−15.98	W (−2.03)
8	2.3	1.4	−20.04	−15.99	I (+4.05)
9	1.2	0.4	−19.77	−17.30	I (+2.47)
Average 1.6 ± 0.6, 0.8 ± 0.5			−22.24 ± 6.5, −18.36 ± 5.1		

BCVA: best-corrected visual acuity; logMAR: logarithm of minimal angle of resolution; MD: mean deviation; VF: visual field; I: improved, N: no change, W: worsened. A change in MD value of 1.0 dB or more was considered an improvement or worsening.

**Table 3:** Changes from baseline in visual acuity and Goldmann perimetry test.

Case	BCVA (logMAR)		Total Degree of VF by the Target of I-4e		Detection of New Isopter *	VF Improvement
	Baseline	After	Baseline	After		
	DSAEK		DSAEK			
1	1.2	0.4	0	20	+	I
2	1.7	0.5	0	81	+	I
3	2	0.15	9	140	+	I
4	1.4	0.7	45	50	−	N
5	2.3	0.7	0	76	+	I
10	1.3	0.8	0	87	+	I
11	2.3	1.3	0	0	+	I
12	2.3	1.7	0	0	−	N
13	2.3	2.3	0	0	−	N
14	1.1	0.7	0	85	+	I
15	2.3	1.0	0	57	+	I
16	1.2	0.4	0	141	+	I
17	1.7	0.7	0	21	+	I
18	2.3	0.8	35	10	−	W
19	0.7	0.4	78	84	−	N

BCVA: best-corrected visual acuity; VF: visual field; DSAEK: Descemet stripping automated endothelial keratoplasty; logMAR: logarithm of minimal angle of resolution. I: improved; N: no change; W: worsened. * The new detection of the smaller or darker isopter was defined as improvement of visual field sensitivity.

## Data Availability

No new data were created or analyzed in this study. Data sharing is not applicable to this article.
